# The HIE-FDTD Method for Simulating Dispersion Media Represented by Drude, Debye, and Lorentz Models

**DOI:** 10.3390/nano13071180

**Published:** 2023-03-26

**Authors:** Juan Chen, Chunhui Mou

**Affiliations:** 1 School of Information and Communications Engineering, Xi’an Jiaotong University, Xi’an 710049, China; 2 Shenzhen Research School, Xi’an Jiaotong University, Shenzhen 518057, China

**Keywords:** convolutional perfectly matched layer (CPML), dispersion media, hybrid implicit–explicit finite-difference time-domain (HIE-FDTD)

## Abstract

The hybrid implicit–explicit finite-difference time-domain (HIE-FDTD) method is a weakly conditionally stable finite-difference time-domain (FDTD) method that has attracted much attention in recent years. However due to the dispersion media such as water, soil, plasma, biological tissue, optical materials, etc., the application of the HIE-FDTD method is still relatively limited. Therefore, in this paper, the HIE-FDTD method was extended to solve typical dispersion media by combining the Drude, Debye, and Lorentz models with hybrid implicit–explicit difference techniques. The advantage of the presented method is that it only needs to solve a set of equations, and then different dispersion media including water, soil, plasma, biological tissue, and optical materials can be analyzed. The convolutional perfectly matched layer (CPML) boundary condition was introduced to truncate the computational domain. Numerical examples were used to validate the absorbing performance of the CPML boundary and prove the accuracy and computational efficiency of the dispersion HIE-FDTD method proposed in this paper. The simulated results showed that the dispersion HIE-FDTD method could not only obtain accurate calculation results, but also had a much higher computational efficiency than the finite-difference time-domain (FDTD) method.

## 1. Introduction

Nanomaterials are widely used in the field of electromagnetism because of their special structure. First, nanomaterials can be used as wave-absorbing materials. On one hand, nano-absorbing materials are widely used on stealth aircraft, tanks, and other military equipment to reduce their probability of detection and destruction and improve the survivability of these targets. On the other hand, nano-absorbing materials are also widely used in daily life such as in the windshields of vehicles and the doors and windows of some public places. Second, nanomaterials have been intensively researched in the field of electromagnetic radiation protection. With the wide application of electromagnetic radiation in communication, transportation, medical, military, and other fields, the human living environment is increasingly polluted by electromagnetic radiation. Research has shown that electromagnetic radiation can affect or even damage biological tissues through various mechanisms. Nanomaterials for electromagnetic radiation protection applications are mainly divided into two categories. One includes radiation protection nanomaterials such as nano-metal materials, nan-oxide materials, and so on. The other is nano protective fabric. In addition, graphene, as the thinnest and strongest new nanomaterial, has been widely used in microwave device design. Graphene is a two-dimensional material made of carbon atoms, so it is only one atom thick. Furthermore, graphene has unique electrical properties, excellent mechanical properties, and thermal stability. Nanomaterials are relatively difficult and costly to prepare, so the co-simulation of nanomaterials and electromagnetic devices is an important prior mean in engineering applications.

In recent years, various electromagnetic calculation methods have vigorously developed. Among them, the finite-difference time-domain (FDTD) method is one of the most important methods [1]. However, due to the limitation of the Courant–Friedrich–Levy (CFL) condition [2], the traditional FDTD method is limited by the minimum grid of the computational space, and its computational efficiency is very low when it is used to simulate electromagnetic problems with fine structures. Especially in the co-simulation of nanomaterials and electromagnetic devices such as the simulation of graphene-based devices, the presence of nanomaterials makes the minimum spatial grid at the nanometer level, which leads to a very small time step size in the FDTD method. At this point, the computational efficiency of the FDTD method is greatly reduced. In addition, although there are commercial software such as CST Studio Suit and Ansys HFSS, which can simulate graphene, they all equate graphene as an impedance surface. Therefore, the physical properties of the graphene interlayer cannot be analyzed. Recently, scholars have developed a hybrid implicit–explicit finite-difference time-domain (HIE-FDTD) method [3,4,5,6,7,8]. The time step size in the HIE-FDTD method is not related to the minimum grid in the computational domain, so the HIE-FDTD method has a much higher computational efficiency than the traditional FDTD method in the simulation of fine objects. There are many applications of the HIE-FDTD method in the simulation of antennas [9], absorbers [10,11], enclosures [12], couplers [13], etc. Especially in the simulation of graphene-based devices, the computation time of the HIE-FDTD method is much shorter than that of the traditional FDTD method [14,15,16,17,18,19]. In [14], a graphene-based polarizer was simulated by using the HIE-FDTD method. The grid size in the graphene sheet was 0.25 nm, and the mesh size in the other part of the computation domain was 1 μm. The calculation time of the HIE-FDTD method was only 1/16,500 of that of the traditional FDTD method with guaranteed calculation accuracy. In [15], Ramadan provided a detailed discussion of the implementation of the HIE-FDTD method in graphene and demonstrated the time stability of the algorithm by using the root-locus method. In [18], Chen et al. analyzed the shielding effectiveness of a graphene-coated shielding sheet by adopting the HIE-FDTD method. Numerical results showed that the shielding effect of the sheets could be improved by controlling the chemical potential of the graphene, and the shielding performance of the sheet was strongly related to the inter-band conductivity of the graphene.

With the rapid development and application of new electromagnetic materials, the propagation mechanism of electromagnetic waves in dispersive media has been continuously explored by scholars. The application of electromagnetic wave propagation characteristics in dispersive media in the fields of global navigation, aerospace, microwave remote sensing, radar technology, target stealth, electronic components, and circuit design has attracted a lot of attention. There are fine structure targets in the problem of electromagnetic wave propagation in dispersive media such as multi-scale plasma dispersive media containing fine structures, or extremely thin graphene dispersive material. If the FDTD method is used to calculate these problems, the computational efficiency is very low. Although the HIE-FDTD method has been widely used in the simulation of objects with homogeneous, linear, and isotropic media, for complex media such as water, soil, plasma, biological tissue, and optical materials, the application of the HIE-FDTD method is still relatively limited. This is because the dielectric constant of these media is mostly related to the operating frequency, that is, the media have dispersive properties. Dispersive media can usually be represented by three typical models, namely, the Drude model, Debye model, and Lorentz model. It is often difficult to simulate these dispersion models by using the HIE-FDTD method. In [20], a frequency-dependent HIE-FDTD method was developed to compute the incidence of a microwave at a planar air–water interface over a wide frequency band. In [21], the artificial anisotropy one-step leapfrog HIE-FDTD method was extended to analyze problems involving Debye media based on the auxiliary differential equation (ADE) formulation. Although in these works they proved that the HIE-FDTD method had high accuracy and efficiency when used in the simulation for dispersion media, these works cannot be used to calculate the objects in the open domain due to the lack of absorbing boundary conditions. Furthermore, the existing HIE-FDTD method can only analyze the Drude, Debye, and Lorentz models separately, and cannot simulate the target composed of two or more dispersion models.

In this paper, we combined the Drude model, Debye model, and Lorentz model with Maxwell’s equations, and used auxiliary differential equations to obtain the unified iterative formula of the HIE-FDTD method for these three dispersion models. Thus, the presented method can be used to calculate water, soil, plasma, biological tissue, optical materials, etc. In addition, we introduced the convolutional perfectly matched layer (CPML) boundary condition in the dispersion HIE-FDTD method, so the method can not only simulate closed-domain problems, but also calculate open-domain problems. The high absorbing performance of the CPML was verified, and numerical examples were used to prove the accuracy and computational efficiency of the dispersion HIE-FDTD method. This proves that the dispersion HIE-FDTD not only had accurate calculated results, but also had a much higher computational efficiency than the conventional FDTD method.

## 2. Methods and Formulations

The permittivity of many media varies with frequency such as plasma, water, biological tissue, radar absorbing materials, etc. Such media are called dispersive media. In this paper, it was assumed that the magnetic constitutive parameters of these media were independent of the frequency, and only the dielectric coefficients were related to the frequency. The frequency constitutive relation is

(1) $$\vec{D}\left(\omega \right)=\varepsilon \left(\omega \right)\vec{E}\left(\omega \right)=\varepsilon_{0}\left[\varepsilon_{\infty }+\chi \left(\omega \right)\right]\vec{E}\left(\omega \right)=\varepsilon_{0}\varepsilon_{\infty }\vec{E}\left(\omega \right)+\varepsilon_{0}\chi \left(\omega \right)\vec{E}\left(\omega \right)$$

where $\varepsilon_{0}$ is the vacuum permittivity; $\varepsilon_{\infty }$ is the relative permittivity at infinite frequency; *χ*(*ω*) is called the polarizability function.

In dispersive media, Maxwell’s time-domain curl equation is written as

(2) $$\nabla \times \vec{H}=\frac{\partial \vec{D}}{\partial t}+\sigma \vec{E}=\varepsilon_{0}\varepsilon_{\infty }\frac{\partial \vec{E}}{\partial t}+\sigma \vec{E}+{\vec{J}}_{p}$$

where *σ* is the conductivity of the medium. If the medium is lossless, *σ* equals zero. ${\vec{J}}_{p}$ is the polarization current, and its frequency domain form is

(3) $${\vec{J}}_{p}\left(\omega \right)=j\omega \varepsilon_{0}\chi \left(\omega \right)\vec{E}\left(\omega \right)$$

Using the centered finite-difference expression for the time derivative in Equation (2), we obtain

(4) $${\left[\nabla \times \vec{H}\right]}^{n+1/2}=\varepsilon_{0}\varepsilon_{\infty }\frac{{\vec{E}}^{n+1}-{\vec{E}}^{n}}{\Delta t}+\sigma \frac{{\vec{E}}^{n+1}+{\vec{E}}^{n}}{2}+\frac{{\vec{J}}_{p}^{n+1}+{\vec{J}}_{p}^{n}}{2}$$

Therefore, for different dispersive media, we only need to find the relationship between their polarization current and electric field, and then substitute this relationship into Equation (4). Finally the calculation formula of each electric field component can be obtained by using centered finite-difference expressions for the space derivatives in Equation (4). There are three typical frequency models for dispersive media: the Drude model, Debye model, and Lorentz model. Next, we will analyze these models, respectively.

### 2.1. Drude Model

The Drude model was proposed by Paul Drude in 1900 to explain the transport properties of electrons in matter, especially in metal. This model is based on four assumptions. (1) There is no interaction or collision between free electrons, and the electrons can move independently of each other. (2) The ions in the crystal are stationary, while the electrons can move freely, and there is no electrostatic interaction between electrons and ions. (3) There are collisions between electrons and ions, and the collision is instantaneous. The motion velocity of electrons after the collision is only related to temperature, and has nothing to do with the velocity of the electrons before the collision. Through this collision, the electrons reach a thermal equilibrium with the surrounding environment. (4) The electron collisions follow the Poisson process and the average time interval between two collisions is called relaxation time. The permittivity of the Drude model is expressed as

(5) $$\varepsilon \left(\omega \right)=\varepsilon_{0}\left[\varepsilon_{\infty }+\chi \left(\omega \right)\right]=\varepsilon_{0}\varepsilon_{\infty }+\varepsilon_{0}\frac{\omega_{p}^{2}}{\omega \left(\omega -j\nu_{c}\right)}$$

where $\varepsilon_{\infty }$ is the relative permittivity at infinite frequency; $\nu_{c}$ is the collision frequency; *τ* is the relaxation time and $\tau =1/\nu_{c}$; $\omega_{p}$ is the Drude frequency, and the polarizability is

(6) $$\chi \left(\omega \right)=\frac{\omega_{p}^{2}}{\omega \left(\omega -j\nu_{c}\right)}$$

The Drude model is often used to describe the dispersion characteristics of plasmas, metals, etc.

By substituting Equation (6) into Equation (3), we obtain

(7) $${\vec{J}}_{p}\left(\omega \right)=j\omega \varepsilon_{0}\frac{\omega_{p}^{2}}{\omega \left(\omega -j\nu_{c}\right)}\vec{E}\left(\omega \right)$$

Equation (7) is the frequency-domain expression between the polarization current and the electric field of the Drude model. An efficient means to obtain the time-domain expression between the polarization current and the electric field is to first multiply both sides of Equation (17) by $\omega \left(\omega -j\nu_{c}\right)$. This gives

(8) $$\omega^{2}{\vec{J}}_{p}\left(\omega \right)-j\omega \nu_{c}{\vec{J}}_{p}\left(\omega \right)=j\omega \varepsilon_{0}\omega_{p}^{2}\vec{E}\left(\omega \right)$$

By applying the transition relation jω→∂/∂t in Equation (8), we can obtain the time-domain expression of Equation (8) as

(9) $$\frac{\partial^{2}{\vec{J}}_{p}}{\partial t}+\nu_{c}\frac{\partial {\vec{J}}_{p}}{\partial t}=-\varepsilon_{0}\omega_{p}^{2}\frac{\partial \vec{E}}{\partial t}$$

Integrating both sides of Equation (9) over time and ignoring the constant term after integration, we obtain

(10) $$\frac{\partial {\vec{J}}_{p}}{\partial t}+\nu_{c}{\vec{J}}_{p}=-\varepsilon_{0}\omega_{p}^{2}\vec{E}$$

Using the centered finite-difference expression for the time derivative in Equation (10), we obtain

(11) $$\frac{{\vec{J}}_{p}^{n+1}-{\vec{J}}_{p}^{n}}{\Delta t}+\nu_{c}\frac{{\vec{J}}_{p}^{n+1}+{\vec{J}}_{p}^{n}}{2}=-\varepsilon_{0}\omega_{p}^{2}\frac{{\vec{E}}^{n+1}+{\vec{E}}^{n}}{2}$$

Solving Equation (11), we obtain

(12) $${\vec{J}}_{p}^{n+1}=k_{p}{\vec{J}}_{p}^{n}+\beta_{p}\left({\vec{E}}^{n+1}+{\vec{E}}^{n}\right)$$

where $k_{p}=\frac{2-\nu_{c}\Delta t}{2+\nu_{c}\Delta t}$, $\beta_{p}=-\frac{\varepsilon_{0}\omega_{p}^{2}\Delta t}{2+\nu_{c}\Delta t}$. Equation (12) is the finite-difference equation between the polarization current and the electric field of the Drude model.

Substituting Equation (12) into Equation (4), we obtain

(13) $${\left[\nabla \times \vec{H}\right]}^{n+1/2}=\varepsilon_{0}\varepsilon_{\infty }\frac{{\vec{E}}^{n+1}-{\vec{E}}^{n}}{\Delta t}+\sigma \frac{{\vec{E}}^{n+1}+{\vec{E}}^{n}}{2}+\frac{k_{p}}{2}{\vec{J}}_{p}^{n}+\frac{\beta_{p}}{2}\left({\vec{E}}^{n+1}+{\vec{E}}^{n}\right)+\frac{{\vec{J}}_{p}^{n}}{2}$$

Equation (13) can be simplified, and it becomes

(14) $$\frac{\Delta t}{\varepsilon_{eff}}{\left[\nabla \times \vec{H}\right]}^{n+1/2}=D_{e1}{\vec{E}}^{n+1}-D_{e2}{\vec{E}}^{n}-D_{e3}{\vec{J}}_{p}^{n}$$

where $\varepsilon_{eff}=\varepsilon_{0}\varepsilon_{\infty }$, $D_{e1}=1+\frac{\sigma }{2}\frac{\Delta t}{\varepsilon_{eff}}+\frac{\beta_{p}}{2}\frac{\Delta t}{\varepsilon_{eff}}$, $D_{e2}=1-\frac{\sigma }{2}\frac{\Delta t}{\varepsilon_{eff}}-\frac{\beta_{p}}{2}\frac{\Delta t}{\varepsilon_{eff}}$, $D_{e3}=-\frac{k_{p}+1}{2}\frac{\Delta t}{\varepsilon_{eff}}$.

### 2.2. Debye Model

Under the action of an applied electric field, the dielectric will be electrically polarized. Electric polarization is a microscopic process, but it can be comprehensively reflected by macroscopic physical quantity. This quantity is the relative permittivity. In addition, polarization relaxation is an important concept in the analysis of electric polarization phenomenon. Polarization relaxation means that when the external electric field acts on the dielectric, it takes a certain period of time for the polarization intensity ***P*** to reach a steady state value. The Debye model considers the polarization relaxation phenomenon in the polarization process and its permittivity is expressed as

(15) $$\varepsilon \left(\omega \right)=\varepsilon_{0}\left[\varepsilon_{\infty }+\chi \left(\omega \right)\right]=\varepsilon_{0}\varepsilon_{\infty }+\varepsilon_{0}\frac{j\nu_{c}\left(\varepsilon_{s}-\varepsilon_{\infty }\right)}{j\nu_{c}-\omega }$$

where $\varepsilon_{s}$ is the relative permittivity at static or zero frequency; $\nu_{c}$ is the collision frequency; *τ* is the relaxation time; $\tau =1/\nu_{c}$. The polarizability χ(ω) in the Debye model is

(16) $$\chi \left(\omega \right)=\frac{\Delta \varepsilon }{1+j\omega \tau }$$

where $\Delta \varepsilon =\varepsilon_{s}-\varepsilon_{\infty }$. The Debye model is often used to describe the dispersion characteristics of media such as soil, water, and human tissue.

By substituting Equation (16) into Equation (3), we obtain

(17) $${\vec{J}}_{p}\left(\omega \right)=j\omega \varepsilon_{0}\chi \left(\omega \right)\vec{E}\left(\omega \right)=j\omega \varepsilon_{0}\frac{\Delta \varepsilon }{1+j\omega \tau }\vec{E}\left(\omega \right)$$

Equation (17) is the frequency-domain expression between the polarization current and the electric field of the Debye model. An efficient means to obtain the time-domain expression between the polarization current and the electric field is to first multiply both sides of Equation (17) by 1+jωτ. This gives

(18) $${\vec{J}}_{p}\left(\omega \right)+j\omega \tau {\vec{J}}_{p}\left(\omega \right)=j\omega \varepsilon_{0}\Delta \varepsilon \vec{E}\left(\omega \right)$$

By applying the transition relation jω→∂/∂t in Equation (18), we obtain

(19) $${\vec{J}}_{p}+\tau \frac{\partial {\vec{J}}_{p}}{\partial t}=\varepsilon_{0}\Delta \varepsilon \frac{\partial \vec{E}}{\partial t}$$

Using the centered finite-difference expression for the time derivative in Equation (19), we obtain

(20) $$\frac{{\vec{J}}_{p}^{n+1}+{\vec{J}}_{p}^{n}}{2}+\tau \frac{{\vec{J}}_{p}^{n+1}-{\vec{J}}_{p}^{n}}{\Delta t}=\varepsilon_{0}\Delta \varepsilon \frac{{\vec{E}}^{n+1}-{\vec{E}}^{n}}{\Delta t}$$

Equation (20) can also be expressed in the following form

(21) $${\vec{J}}_{p}^{n+1}=k_{p1}{\vec{J}}_{p}^{n}+\beta_{p1}\left(\frac{{\vec{E}}^{n+1}-{\vec{E}}^{n}}{\Delta t}\right)$$

where $k_{p1}=\frac{2\tau -\Delta t}{2\tau +\Delta t}$, $\beta_{p1}=\frac{2\varepsilon_{0}\Delta \varepsilon \Delta t}{2\tau +\Delta t}$. Equation (21) is the finite-difference equation between the polarization current and the electric field of the Debye model.

Substituting Equation (21) into Equation (4), we obtain

(22) $${\left[\nabla \times \vec{H}\right]}^{n+1/2}=\varepsilon_{0}\varepsilon_{\infty }\frac{{\vec{E}}^{n+1}-{\vec{E}}^{n}}{\Delta t}+\sigma \frac{{\vec{E}}^{n+1}+{\vec{E}}^{n}}{2}+\frac{k_{p1}}{2}{\vec{J}}_{p}^{n}+\frac{\beta_{p1}}{2}\frac{\left({\vec{E}}^{n+1}-{\vec{E}}^{n}\right)}{\Delta t}+\frac{{\vec{J}}_{p}^{n}}{2}$$

Equation (22) can be simplified, and it becomes

(23) $$\frac{\Delta t}{\varepsilon_{eff}}{\left[\nabla \times \vec{H}\right]}^{n+1/2}=D_{e4}{\vec{E}}^{n+1}-D_{e5}{\vec{E}}^{n}-D_{e6}{\vec{J}}_{p}^{n}$$

where $\varepsilon_{eff}=\varepsilon_{0}\varepsilon_{\infty }$, $D_{e4}=1+\frac{\sigma }{2}\frac{\Delta t}{\varepsilon_{eff}}+\frac{\beta_{p1}}{2\varepsilon_{eff}}$, $D_{e5}=1-\frac{\sigma }{2}\frac{\Delta t}{\varepsilon_{eff}}+\frac{\beta_{p1}}{2\varepsilon_{eff}}$, $D_{e6}=-\frac{k_{p1}+1}{2}\frac{\Delta t}{\varepsilon_{eff}}$.

### 2.3. Lorentz Model

In the Lorentz model, it is considered that the electron is no longer free and unrestrained. The electron and the ionic are bound to each other by the Coulomb force, and the motion of the electron is simple harmonic vibration. Considering that the linear oscillator has elastic restoring force and damping force, the expression for the permittivity of the Lorentz model is

(24) $$\varepsilon \left(\omega \right)=\varepsilon_{0}\left[\varepsilon_{\infty }+\chi \left(\omega \right)\right]=\varepsilon_{0}\varepsilon_{\infty }+\varepsilon_{0}\frac{\left(\varepsilon_{s}-\varepsilon_{\infty }\right)\omega_{0}^{2}}{\omega_{0}^{2}-\omega^{2}+2j\omega \nu_{c}}$$

where $\varepsilon_{s}$ is the relative permittivity at static or zero frequency; $\varepsilon_{\infty }$ is the relative permittivity at infinite frequency; $\omega_{0}$ is the natural frequency of the oscillator; $\nu_{c}$ is the collision frequency; *τ* is the relaxation time and $\tau =1/\nu_{c}$.

The polarizability χ(ω) of Lorentz model is

(25) $$\chi \left(\omega \right)=\frac{\left(\varepsilon_{s}-\varepsilon_{\infty }\right)\omega_{0}^{2}}{\omega_{0}^{2}-\omega^{2}+2j\omega \nu_{c}}$$

By substituting Equation (25) into Equation (3), we obtain

(26) $${\vec{J}}_{p}\left(\omega \right)=j\omega \varepsilon_{0}\chi \left(\omega \right)\vec{E}\left(\omega \right)=j\omega \varepsilon_{0}\frac{\Delta \varepsilon \omega_{0}^{2}}{\omega_{0}^{2}-\omega^{2}+2j\omega \nu_{c}}\vec{E}\left(\omega \right)$$

where $\Delta \varepsilon =\varepsilon_{s}-\varepsilon_{\infty }$. Equation (26) is the frequency-domain expression between the polarization current and the electric field of the Lorentz model. An efficient means to obtain the time-domain expression between the polarization current and the electric field is to first multiply both sides of Equation (26) by $\omega_{0}^{2}-\omega^{2}+2j\omega \nu_{c}$. This obtains

(27) $$\omega_{0}^{2}{\vec{J}}_{p}\left(\omega \right)-\omega^{2}{\vec{J}}_{p}\left(\omega \right)+2j\omega \nu_{c}{\vec{J}}_{p}\left(\omega \right)=j\omega \varepsilon_{0}\Delta \varepsilon \omega_{0}^{2}\vec{E}\left(\omega \right)$$

By applying the transition relation jω→∂/∂t in Equation (27), we obtain

(28) $$\omega_{0}^{2}{\vec{J}}_{p}+\frac{\partial^{2}{\vec{J}}_{p}}{\partial t^{2}}+2\nu_{c}\frac{\partial {\vec{J}}_{p}}{\partial t}=\varepsilon_{0}\Delta \varepsilon \omega_{0}^{2}\frac{\partial \vec{E}}{\partial t}$$

Using the centered finite-difference expression for the time derivative in Equation (28), we obtain

(29) $$\omega_{0}^{2}{\vec{J}}_{p}^{n}+2\nu_{c}\frac{{\vec{J}}_{p}^{n+1}-{\vec{J}}_{p}^{n-1}}{2\Delta t}+\frac{{\vec{J}}_{p}^{n+1}-2{\vec{J}}_{p}^{n}+{\vec{J}}_{p}^{n-1}}{\Delta t^{2}}=\varepsilon_{0}\Delta \varepsilon \omega_{0}^{2}\frac{{\vec{E}}^{n+1}-{\vec{E}}^{n-1}}{2\Delta t}$$

Equation (29) can also be expressed in the following form

(30) $${\vec{J}}_{p}^{n+1}=k_{p2}{\vec{J}}_{p}^{n}+k_{p3}{\vec{J}}_{p}^{n-1}+\beta_{p2}\left(\frac{{\vec{E}}^{n+1}-{\vec{E}}^{n-1}}{2\Delta t}\right)$$

where $k_{p2}=\frac{2-\omega_{0}^{2}{\left(\Delta t\right)}^{2}}{1+\nu_{c}\Delta t}$, $k_{p3}=\frac{\nu_{c}\Delta t-1}{1+\nu_{c}\Delta t}$, $\beta_{p2}=\frac{\varepsilon_{0}\Delta \varepsilon \omega_{0}^{2}{\left(\Delta t\right)}^{2}}{1+\nu_{c}\Delta t}$. Equation (30) is the finite-difference equation between the polarization current and the electric field of the Lorentz model.

By substituting Equation (30) into Equation (4), we obtain

(31) $${\left[\nabla \times \vec{H}\right]}^{n+1/2}=\varepsilon_{0}\varepsilon_{\infty }\frac{{\vec{E}}^{n+1}-{\vec{E}}^{n}}{\Delta t}+\sigma \frac{{\vec{E}}^{n+1}+{\vec{E}}^{n}}{2}+\frac{k_{p2}}{2}{\vec{J}}_{p}^{n}+\frac{k_{p3}}{2}{\vec{J}}_{p}^{n-1}+\frac{\beta_{p2}}{2}\frac{{\vec{E}}^{n+1}-{\vec{E}}^{n-1}}{2\Delta t}+\frac{{\vec{J}}_{p}^{n}}{2}$$

Equation (31) can be simplified and becomes

(32) $$\frac{\Delta t}{\varepsilon_{eff}}{\left[\nabla \times \vec{H}\right]}^{n+1/2}=D_{e7}{\vec{E}}^{n+1}-D_{e8}{\vec{E}}^{n}-D_{e9}{\vec{E}}^{n-1}-D_{e10}{\vec{J}}_{p}^{n}+D_{e11}{\vec{J}}_{p}^{n-1}$$

where $\varepsilon_{eff}=\varepsilon_{0}\varepsilon_{\infty }$, $D_{e7}=1+\frac{\sigma }{2}\frac{\Delta t}{\varepsilon_{eff}}+\frac{\beta_{p2}}{4\varepsilon_{eff}}$, $D_{e8}=1-\frac{\sigma }{2}\frac{\Delta t}{\varepsilon_{eff}}$, $D_{e9}=\frac{\beta_{p2}}{4\varepsilon_{eff}}$, $D_{10}=-\frac{k_{p2}+1}{2}\frac{\Delta t}{\varepsilon_{eff}}$, $D_{e11}=\frac{\Delta t}{\varepsilon_{eff}}\frac{k_{p3}}{2}$.

### 2.4. Standard Model for the Drude, Debye, and Lorentz Models

Based on Equation (14), Equation (23) and Equation (32), the magnetic field curl equation in the time-domain for the Drude, Debye, and Lorentz models can be described as follows:

(33) $$\frac{\Delta t}{\varepsilon_{eff}}{\left[\nabla \times \vec{H}\right]}^{n+1/2}=A_{1}{\vec{E}}^{n+1}-A_{2}{\vec{E}}^{n}-A_{3}{\vec{E}}^{n-1}-A_{4}{\vec{J}}_{p}^{n}+A_{5}{\vec{J}}_{p}^{n-1}$$

where $\varepsilon_{eff}=\varepsilon_{0}\varepsilon_{\infty }$.

For the Drude model, it has *A*_1_ = *D*_e1_, *A*_2_ = *D*_e2_, *A*_3_ = 0, *A*_4_ = *D*_e3_, and *A*_5_ = 0.

For the Debye model, it has *A*_1_ = *D*_e4_, *A*_2_ = *D*_e5_, *A*_3_ = 0, *A*_4_ = *D_e_*_6_, and *A*_5_ = 0.

For the Lorentz model, it has *A*_1_ = *D_e_*_7_, *A*_2_ = *D_e_*_8_, *A*_3_ = *D_e_*_9_, *A*_4_ = *D_e_*_10_, and *A*_5_ = *D_e_*_11_.

Based on Equation (12), Equation (21), and Equation (30), the polarization current for the Drude, Debye, and Lorentz models, respectively, can be calculated as follows:

(34) $${\vec{J}}_{p}^{n+1}=B_{1}{\vec{E}}^{n+1}+B_{2}{\vec{E}}^{n}+B_{3}{\vec{E}}^{n-1}+B_{4}{\vec{J}}_{p}^{n}+B_{5}{\vec{J}}_{p}^{n-1}$$

For the Drude model, it has *B*_1_ = *β_P_*, *B*_2_ = *β_P_*, *B*_3_ = 0, *B*_4_ = *k_P_*, and *B*_5_ = 0.

For the Debye model, it has $B_{1}=\frac{\beta_{p1}}{\Delta t}$, $B_{2}=-\frac{\beta_{p1}}{\Delta t}$, *B*_3_ = 0, *B*_4_ = *k_P_*_1_, and *B*_5_ = 0.

For the Lorentz model, it has $B_{1}=\frac{\beta_{p2}}{2\Delta t}$, *B*_2_ = 0, $B_{3}=-\frac{\beta_{p2}}{2\Delta t}$, *B*_4_ = *k_P_*_2_, and *B*_5_ = *k_P_*_3_.

Equation (34) is the auxiliary differential equation that introduces the dispersion property of the dispersive media into the HIE-FDTD method. 

The electric field curl equation in the time-domain is the same as that in the linear media, namely,

(35) $$\nabla \times \vec{E}=-\mu \frac{\partial \vec{H}}{\partial t}$$

### 2.5. The HIE-FDTD Method in Dispersion Media

Without loss of generality, it is assumed that the fine structure is along the *y* direction, so the hybrid implicit–explicit difference technique was applied to the derivative term ∂y/∂t in the HIE-FDTD method. Thus, according to Equation (33), we obtained the formulas of the three electric field components in dispersive media as

(36) $$\begin{array}{ll} A_{1}E_{y}^{n+1}\left(i,j+\frac{1}{2},k\right) & =A_{2}E_{y}^{n}\left(i,j+\frac{1}{2},k\right)+\\ & \frac{\Delta t}{\varepsilon_{eff}}\frac{1}{\Delta z}\left[H_{x}^{n}\left(i,j+\frac{1}{2},k+\frac{1}{2}\right)-H_{x}^{n}\left(i,j+\frac{1}{2},k-\frac{1}{2}\right)\right]-\\ & \frac{\Delta t}{\varepsilon_{eff}}\frac{1}{\Delta x}\left[H_{z}^{n}\left(i+\frac{1}{2},j+\frac{1}{2},k\right)-H_{z}^{n}\left(i-\frac{1}{2},j+\frac{1}{2},k\right)\right]+\\ & A_{3}E_{y}^{n-1}\left(i,j+\frac{1}{2},k\right)+A_{4}J_{py}^{n}\left(i,j+\frac{1}{2},k\right)-A_{5}J_{py}^{n-1}\left(i,j+\frac{1}{2},k\right) \end{array}$$

(37) $$\begin{array}{ll} A_{1}E_{x}^{n+1}\left(i+\frac{1}{2},j,k\right) & =A_{2}E_{x}^{n}\left(i+\frac{1}{2},j,k\right)-\\ & \frac{\Delta t}{\varepsilon_{eff}}\frac{1}{\Delta z}\left[H_{y}^{n}\left(i+\frac{1}{2},j,k+\frac{1}{2}\right)-H_{y}^{n}\left(i+\frac{1}{2},j,k-\frac{1}{2}\right)\right]+\\ & \frac{\Delta t}{\varepsilon_{eff}}\frac{1}{2\Delta y}\left[H_{z}^{n+1}\left(i+\frac{1}{2},j+\frac{1}{2},k\right)-H_{z}^{n+1}\left(i+\frac{1}{2},j-\frac{1}{2},k\right)\right]+\\ & \frac{\Delta t}{\varepsilon_{eff}}\frac{1}{2\Delta y}\left[H_{z}^{n}\left(i+\frac{1}{2},j+\frac{1}{2},k\right)-H_{z}^{n}\left(i+\frac{1}{2},j-\frac{1}{2},k\right)\right]+\\ & A_{3}E_{x}^{n-1}\left(i+\frac{1}{2},j,k\right)+A_{4}J_{px}^{n}\left(i+\frac{1}{2},j,k\right)-A_{5}J_{px}^{n-1}\left(i+\frac{1}{2},j,k\right) \end{array}$$

(38) $$\begin{array}{ll} A_{1}E_{z}^{n+1}\left(i,j,k+\frac{1}{2}\right) & =A_{2}E_{z}^{n}\left(i,j,k+\frac{1}{2}\right)+\\ & \frac{\Delta t}{\varepsilon_{eff}}\frac{1}{\Delta x}\left[H_{y}^{n}\left(i+\frac{1}{2},j,k+\frac{1}{2}\right)-H_{y}^{n}\left(i-\frac{1}{2},j,k+\frac{1}{2}\right)\right]-\\ & \frac{\Delta t}{\varepsilon_{eff}}\frac{1}{2\Delta y}\left[H_{x}^{n+1}\left(i,j+\frac{1}{2},k+\frac{1}{2}\right)-H_{x}^{n+1}\left(i,j-\frac{1}{2},k+\frac{1}{2}\right)\right]-\\ & \frac{\Delta t}{\varepsilon_{eff}}\frac{1}{2\Delta y}\left[H_{x}^{n}\left(i,j+\frac{1}{2},k+\frac{1}{2}\right)-H_{x}^{n}\left(i,j-\frac{1}{2},k+\frac{1}{2}\right)\right]+\\ & A_{3}E_{z}^{n-1}\left(i,j,k+\frac{1}{2}\right)+A_{4}J_{pz}^{n}\left(i,j,k+\frac{1}{2}\right)-A_{5}J_{pz}^{n-1}\left(i,j,k+\frac{1}{2}\right) \end{array}$$

According to Equation (35), the calculations for the magnetic field are as follows

(39) $$\begin{array}{l} H_{x}^{n+1}\left(i,j+\frac{1}{2},k+\frac{1}{2}\right)=H_{x}^{n}\left(i,j+\frac{1}{2},k+\frac{1}{2}\right)+\frac{\Delta t}{\mu \Delta z}\left[E_{y}^{n+1}\left(i,j+\frac{1}{2},k+1\right)-E_{y}^{n+1}\left(i,j+\frac{1}{2},k\right)\right]-\\ \;\;\;\;\frac{\Delta t}{2\mu \Delta y}\left[E_{z}^{n+1}\left(i,j+1,k+\frac{1}{2}\right)-E_{z}^{n+1}\left(i,j,k+\frac{1}{2}\right)\right]-\frac{\Delta t}{2\mu \Delta y}\left[E_{z}^{n}\left(i,j+1,k+\frac{1}{2}\right)-E_{z}^{n}\left(i,j,k+\frac{1}{2}\right)\right] \end{array}$$

(40) $$\begin{array}{l} H_{y}^{n+1}\left(i+\frac{1}{2},j,k+\frac{1}{2}\right)=H_{y}^{n}\left(i+\frac{1}{2},j,k+\frac{1}{2}\right)-\frac{\Delta t}{\mu \Delta z}\left[E_{x}^{n+1}\left(i+\frac{1}{2},j,k+1\right)-E_{x}^{n+1}\left(i+\frac{1}{2},j,k\right)\right]+\\ \;\;\;\;\;\;\frac{\Delta t}{\mu \Delta x}\left[E_{z}^{n+1}\left(i+1,j,k+\frac{1}{2}\right)-E_{z}^{n+1}\left(i,j,k+\frac{1}{2}\right)\right] \end{array}$$

(41) $$\begin{array}{l} H_{z}^{n+1}\left(i+\frac{1}{2},j+\frac{1}{2},k\right)=H_{z}^{n}\left(i+\frac{1}{2},j+\frac{1}{2},k\right)-\frac{\Delta t}{\mu \Delta x}\left[E_{y}^{n+1}\left(i+1,j+\frac{1}{2},k\right)-E_{y}^{n+1}\left(i,j+\frac{1}{2},k\right)\right]+\\ \;\;\;\frac{\Delta t}{2\mu \Delta y}\left[E_{x}^{n+1}\left(i+\frac{1}{2},j+1,k\right)-E_{x}^{n+1}\left(i+\frac{1}{2},j,k\right)\right]+\frac{\Delta t}{2\mu \Delta y}\left[E_{x}^{n}\left(i+\frac{1}{2},j+1,k\right)-E_{x}^{n}\left(i+\frac{1}{2},j,k\right)\right] \end{array}$$

It can be seen that $E_{x}^{n+1}$ and $E_{z}^{n+1}$ cannot be calculated directly by using Equations (37) and (38) because they all include the unknown magnetic field defined at the same time. Thus, modified equations were derived from the original equations. Substituting Equations (41) and (39) into Equations (37) and (38), respectively, the equations for $E_{x}^{n+1}$ and $E_{z}^{n+1}$ are given as

(42) $$\begin{array}{c} \left[A_{1}+\frac{\Delta t^{2}}{2\varepsilon_{eff}\mu \Delta y^{2}}\right]E_{x}^{n+1}\left(i+\frac{1}{2},j,k\right)-\frac{\Delta t^{2}}{4\varepsilon_{eff}\mu \Delta y^{2}}\left[E_{x}^{n+1}\left(i+\frac{1}{2},j+1,k\right)+E_{x}^{n+1}\left(i+\frac{1}{2},j-1,k\right)\right]=\\ A_{2}E_{x}^{n}\left(i+\frac{1}{2},j,k\right)+\frac{\Delta t}{\varepsilon_{eff}}\frac{1}{\Delta y}\left[H_{z}^{n}\left(i+\frac{1}{2},j+\frac{1}{2},k\right)-H_{z}^{n}\left(i+\frac{1}{2},j-\frac{1}{2},k\right)\right]-\\ \frac{\Delta t^{2}}{2\varepsilon_{eff}\mu \Delta x\Delta y}\left[E_{y}^{n+1}\left(i+1,j+\frac{1}{2},k\right)-E_{y}^{n+1}\left(i,j+\frac{1}{2},k\right)-E_{y}^{n+1}\left(i+1,j-\frac{1}{2},k\right)+E_{y}^{n+1}\left(i,j-\frac{1}{2},k\right)\right]+\\ \frac{\Delta t^{2}}{4\varepsilon_{eff}\mu \Delta y^{2}}\left[E_{x}^{n}\left(i+\frac{1}{2},j+1,k\right)-2E_{x}^{n}\left(i+\frac{1}{2},j,k\right)+E_{x}^{n}\left(i+\frac{1}{2},j-1,k\right)\right]-\\ \frac{\Delta t}{\varepsilon_{eff}}\frac{1}{\Delta z}\left[H_{y}^{n}\left(i+\frac{1}{2},j,k+\frac{1}{2}\right)-H_{y}^{n}\left(i+\frac{1}{2},j,k-\frac{1}{2}\right)\right]\\ +A_{3}E_{x}^{n-1}\left(i+\frac{1}{2},j,k\right)+A_{4}J_{px}^{n}\left(i+\frac{1}{2},j,k\right)-A_{5}J_{px}^{n-1}\left(i+\frac{1}{2},j,k\right) \end{array}$$

(43) $$\begin{array}{c} \left[A_{1}+\frac{\Delta t^{2}}{2\varepsilon_{eff}\mu \Delta y^{2}}\right]E_{z}^{n+1}\left(i,j,k+\frac{1}{2}\right)-\frac{\Delta t^{2}}{4\varepsilon_{eff}\mu \Delta y^{2}}\left[E_{z}^{n+1}\left(i,j+1,k+\frac{1}{2}\right)+E_{z}^{n+1}\left(i,j-1,k+\frac{1}{2}\right)\right]=\\ A_{2}E_{z}^{n}\left(i,j,k+\frac{1}{2}\right)-\frac{\Delta t}{\varepsilon_{eff}}\frac{1}{\Delta y}\left[H_{x}^{n}\left(i,j+\frac{1}{2},k+\frac{1}{2}\right)-H_{x}^{n}\left(i,j-\frac{1}{2},k+\frac{1}{2}\right)\right]-\\ \frac{\Delta t^{2}}{2\varepsilon_{eff}\mu \Delta z\Delta y}\left[E_{y}^{n+1}\left(i,j+\frac{1}{2},k+1\right)-E_{y}^{n+1}\left(i,j+\frac{1}{2},k\right)-E_{y}^{n+1}\left(i,j-\frac{1}{2},k+1\right)+E_{y}^{n+1}\left(i,j-\frac{1}{2},k\right)\right]+\\ \frac{\Delta t^{2}}{4\varepsilon_{eff}\mu \Delta y^{2}}\left[E_{z}^{n}\left(i,j+1,k+\frac{1}{2}\right)-2E_{z}^{n}\left(i,j,k+\frac{1}{2}\right)+E_{z}^{n}\left(i,j-1,k+\frac{1}{2}\right)\right]+\\ \frac{\Delta t}{\varepsilon_{eff}}\frac{1}{\Delta x}\left[H_{y}^{n}\left(i+\frac{1}{2},j,k+\frac{1}{2}\right)-H_{y}^{n}\left(i-\frac{1}{2},j,k+\frac{1}{2}\right)\right]\\ A_{3}E_{z}^{n-1}\left(i,j,k+\frac{1}{2}\right)+A_{4}J_{pz}^{n}\left(i,j,k+\frac{1}{2}\right)-A_{5}J_{pz}^{n-1}\left(i,j,k+\frac{1}{2}\right) \end{array}$$

where the iterative calculations of the polarization current ${\vec{J}}_{px}^{n+1}$, ${\vec{J}}_{py}^{n+1}$, and ${\vec{J}}_{pz}^{n+1}$ refer to Equation (34).

Thus, the iterative process of the HIE-FDTD method in dispersion media are as follows:

(1) Calculate $E_{y}^{n+1}$ by using Equation (36);

(2) Calculate $E_{x}^{n+1}$ and $E_{z}^{n+1}$ by using Equations (42) and (43);

(3) Calculate $H_{x}^{n+1}$, $H_{y}^{n+1}$ and $H_{z}^{n+1}$ by using Equations (39)–(41);

(4) Calculate $J_{px}^{n+1}$, $J_{py}^{n+1}$, and $J_{pz}^{n+1}$ by using Equation (34);

(5) Return to Equation (1).

In [12], the weakly conditional stability of the HIE-FDTD method was well-validated. The time step size of the HIE-FDTD method in the *y* direction was only determined by two spatial increments Δ*x* and Δ*z*, namely,

(44) $$\Delta t\leq 1/\left(c\sqrt{{\left(1/\Delta x\right)}^{2}+{\left(1/\Delta z\right)}^{2}}\right)$$

where *c* is the speed of light in the medium and $c=1/\sqrt{\varepsilon \mu }$. This will significantly reduce the computation time when the simulated structure had fine-scale dimensions in one direction, which will be validated in a later section by using numerical examples. 

### 2.6. The CPML Absorption Boundary in the Dispersion HIE-FDTD Method

To truncate the calculation area of the dispersive medium, the CPML absorption boundary can be applied. The CPML formula does not split the field components and the implementation is convenient [22,23,24].

Taking the calculations of $E_{x}^{n+1}$ and $H_{z}^{n+1}$ as examples, in the CPML absorption boundary, their formulas are expressed as

(45) $$\begin{array}{ll} A_{1}E_{x}^{n+1}\left(i+\frac{1}{2},j,k\right) & =A_{2}E_{x}^{n}\left(i+\frac{1}{2},j,k\right)\\ & -\frac{\Delta t}{\varepsilon_{eff}}\frac{1}{\kappa_{z}\Delta z}\left[H_{y}^{n}\left(i+\frac{1}{2},j,k+\frac{1}{2}\right)-H_{y}^{n}\left(i+\frac{1}{2},j,k-\frac{1}{2}\right)\right]\\ & +\frac{\Delta t}{\varepsilon_{eff}}\frac{1}{2\kappa_{y}\Delta y}\left[H_{z}^{n+1}\left(i+\frac{1}{2},j+\frac{1}{2},k\right)-H_{z}^{n+1}\left(i+\frac{1}{2},j-\frac{1}{2},k\right)\right]\\ & +\frac{\Delta t}{\varepsilon_{eff}}\frac{1}{2\kappa_{y}\Delta y}\left[H_{z}^{n}\left(i+\frac{1}{2},j+\frac{1}{2},k\right)-H_{z}^{n}\left(i+\frac{1}{2},j-\frac{1}{2},k\right)\right]\\ & +A_{3}E_{x}^{n-1}\left(i+\frac{1}{2},j,k\right)+A_{4}J_{px}^{n}\left(i+\frac{1}{2},j,k\right)-A_{5}J_{px}^{n-1}\left(i+\frac{1}{2},j,k\right)\\ & +\frac{\Delta t}{\varepsilon_{eff}}\varphi_{pxy}^{n+1}\left(i+\frac{1}{2},j,k\right)-\frac{\Delta t}{\varepsilon_{eff}}\varphi_{pxz}^{n}\left(i+\frac{1}{2},j,k\right) \end{array}$$

(46) $$\begin{array}{ll} H_{z}^{n+1}\left(i+\frac{1}{2},j+\frac{1}{2},k\right)= & H_{z}^{n}\left(i+\frac{1}{2},j+\frac{1}{2},k\right)-\\ & \frac{\Delta t}{\mu \kappa_{x}\Delta x}\left[E_{y}^{n+1}\left(i+1,j+\frac{1}{2},k\right)-E_{y}^{n+1}\left(i,j+\frac{1}{2},k\right)\right]+\\ & \frac{\Delta t}{2\mu \kappa_{y}\Delta y}\left[E_{x}^{n+1}\left(i+\frac{1}{2},j+1,k\right)-E_{x}^{n+1}\left(i+\frac{1}{2},j,k\right)\right]+\\ & \frac{\Delta t}{2\mu \kappa_{y}\Delta y}\left[E_{x}^{n}\left(i+\frac{1}{2},j+1,k\right)-E_{x}^{n}\left(i+\frac{1}{2},j,k\right)\right]+\\ & \frac{\Delta t}{\mu }\Psi_{mzy}^{n+1}\left(i+\frac{1}{2},j+\frac{1}{2},k\right)-\frac{\Delta t}{\mu }\Psi_{mzx}^{n+1}\left(i+\frac{1}{2},j+\frac{1}{2},k\right) \end{array}$$

By substituting Equation (46) into Equation (45), we obtain

(47) $$\begin{array}{c} \left(A_{1}+2\tau_{1}\right)E_{x}^{n+1}\left(i+\frac{1}{2},j,k\right)-\tau_{1}\left[E_{x}^{n+1}\left(i+\frac{1}{2},j+1,k\right)+E_{x}^{n+1}\left(i+\frac{1}{2},j-1,k\right)\right]=\\ \left(A_{2}-2\tau_{1}\right)E_{x}^{n}(i+\frac{1}{2},j,k)+\tau_{1}\left[E_{x}^{n}\left(i+\frac{1}{2},j+1,k\right)+E_{x}^{n}\left(i+\frac{1}{2},j-1,k\right)\right]\\ +\chi_{1}\left[H_{z}^{n}\left(i+\frac{1}{2},j+\frac{1}{2},k\right)-H_{z}^{n}\left(i+\frac{1}{2},j-\frac{1}{2},k\right)\right]\\ +\chi_{2}\left[E_{y}^{n+1}\left(i+1,j+\frac{1}{2},k\right)-E_{y}^{n+1}\left(i,j+\frac{1}{2},k\right)-E_{y}^{n+1}\left(i+1,j-\frac{1}{2},k\right)+E_{y}^{n+1}\left(i,j-\frac{1}{2},k\right)\right]\\ +\chi_{3}\left[H_{y}^{n}\left(i+\frac{1}{2},j,k+\frac{1}{2}\right)-H_{y}^{n}\left(i+\frac{1}{2},j,k-\frac{1}{2}\right)\right]\\ +A_{3}E_{x}^{n-1}\left(i+\frac{1}{2},j,k\right)+A_{4}J_{px}^{n}\left(i+\frac{1}{2},j,k\right)-A_{5}J_{px}^{n-1}\left(i+\frac{1}{2},j,k\right)+\beta \end{array}$$

where $\tau_{1}=\frac{\Delta t^{2}{\left(1/\kappa_{y}+a_{y}\right)}^{2}}{4\varepsilon_{eff}\mu \Delta y^{2}}$, $\chi_{2}=-\frac{\Delta t^{2}\left(1/\kappa_{y}+a_{y}\right)\left(1/\kappa_{x}+a_{x}\right)}{2\mu \varepsilon_{eff}\Delta x\Delta y}$, $\chi_{1}=\frac{\Delta t\left(1/\kappa_{y}+a_{y}\right)}{\varepsilon_{eff}\Delta y}$, $\chi_{3}=-\frac{\Delta t}{\varepsilon_{eff}\Delta z\kappa_{z}}$.

$$\begin{array}{ll} \beta & =\frac{b_{y}\Delta t}{\varepsilon_{eff}}\varphi_{pxy}^{n}\left(i+\frac{1}{2},j,k\right)-\frac{\Delta t}{\varepsilon_{eff}}\varphi_{pxz}^{n}\left(i+\frac{1}{2},j,k\right)\\ & +\frac{\Delta t^{2}}{2\varepsilon_{eff}\mu \Delta y}\left(1/\kappa_{y}+a_{y}\right)\left[b_{y}\varphi_{mzy}^{n}\left(i+\frac{1}{2},j+\frac{1}{2},k\right)-b_{y}\varphi_{mzy}^{n}\left(i+\frac{1}{2},j-\frac{1}{2},k\right)\right]\\ & -\frac{\Delta t^{2}}{2\varepsilon_{eff}\mu \Delta y}\left(1/\kappa_{y}+a_{y}\right)b_{x}\left[\varphi_{mzx}^{n}\left(i+\frac{1}{2},j+\frac{1}{2},k\right)-\varphi_{mzx}^{n}\left(i+\frac{1}{2},j-\frac{1}{2},k\right)\right] \end{array}.$$

Auxiliary variables *φ* in CPML absorbing boundary are calculated as follows

(48) $$\varphi_{pxy}^{n+1}\left(i+\frac{1}{2},j,k\right)=b_{y}\varphi_{pxy}^{n}\left(i+\frac{1}{2},j,k\right)+a_{y}\frac{H_{z}^{n+1}\left(i+\frac{1}{2},j+\frac{1}{2},k\right)-H_{z}^{n+1}\left(i+\frac{1}{2},j-\frac{1}{2},k\right)}{2\Delta y}+a_{y}\frac{H_{z}^{n}\left(i+\frac{1}{2},j+\frac{1}{2},k\right)-H_{z}^{n}\left(i+\frac{1}{2},j-\frac{1}{2},k\right)}{2\Delta y}$$

(49) $$\varphi_{pxz}^{n}\left(i+\frac{1}{2},j,k\right)=b_{z}\varphi_{pxz}^{n-1}\left(i+\frac{1}{2},j,k\right)+a_{z}\frac{H_{y}^{n}\left(i+\frac{1}{2},j,k+\frac{1}{2}\right)-H_{y}^{n}\left(i+\frac{1}{2},j,k-\frac{1}{2}\right)}{\Delta z}$$

(50) $$\varphi_{mzx}^{n+1}\left(i+\frac{1}{2},j+\frac{1}{2},k\right)=b_{x}\varphi_{mzx}^{n}\left(i+\frac{1}{2},j+\frac{1}{2},k\right)+a_{x}\frac{E_{y}^{n+1}\left(i+1,j+\frac{1}{2},k\right)-E_{y}^{n+1}\left(i,j+\frac{1}{2},k\right)}{\Delta x}$$

(51) $$\varphi_{mzy}^{n+1}\left(i+\frac{1}{2},j+\frac{1}{2},k\right)=b_{y}\varphi_{mzy}^{n}\left(i+\frac{1}{2},j+\frac{1}{2},k\right)+a_{y}\frac{E_{x}^{n+1}\left(i+\frac{1}{2},j+1,k\right)-E_{x}^{n+1}\left(i+\frac{1}{2},j,k\right)}{2\Delta y}+a_{y}\frac{E_{x}^{n}\left(i+\frac{1}{2},j+1,k\right)-E_{x}^{n}\left(i+\frac{1}{2},j,k\right)}{2\Delta y}$$

The values of parameters $\kappa_{m},\;a_{m}$, and $b_{m}$ (*m* = *x*, *y*, *z*) in the above calculations refer to the formulas in [22]. The updating equations of other field components in the CPML absorbing boundary can be derived in the same way.

In the region without the CPML boundary, $\kappa_{m}=1,\;a_{m}=0,\;b_{m}=0$, at which time Equations (45) and (46) degenerate to Equations (37) and (41), respectively. This means that Equations (45) and (46) are applicable to both the CPML region and non-CPML region.

## 3. Numerical Validations

### 3.1. Performance of Absorbing Boundary

To illustrate the CPML termination performance, the simulation of radiation from a small current source in a dispersive space was studied. The total computational domain was 45 × 45 × 45 cells, and a uniform mesh with a cell size Δ*x* = Δ*y* = Δ*z* = 0.015 m was applied. The volume of the dispersive media was 0.075 m × 0.075 m×0.075 m and located in the center of the computational domain. The parameters of the dispersive media are shown in Table 1. A 10-cell thick CPML boundary, which is ten cells far from the dispersive media, was used to terminate all six sides of the lattice. 

**Figure 1:**
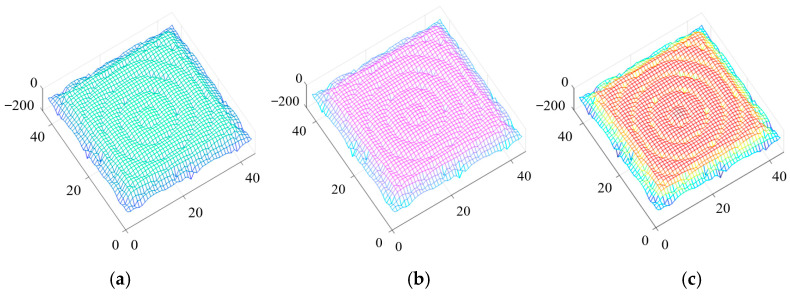
The field distribution in the computational domain calculated by using the dispersion HIE-FDTD method: (**a**) Drude model; (**b**) Debye model; (**c**) Lorentz model.

Figure 1 shows the steady field distribution in the computational domain calculated by using the dispersion HIE-FDTD method. Here, a point source with a frequency of 2 GHz was located in the center of the dispersive media. The time step size was selected as $\Delta t=1/\left(c\sqrt{{\left(1/\Delta x\right)}^{2}+{\left(1/\Delta z\right)}^{2}}\right)$ = 35 ps, which was the maximum time step to satisfy the time stability condition in the HIE-FDTD method. From Figure 1, we can see that in all three models, the radiation was annular distribution, and there was no obvious reflection from the absorbing boundary into the computational domain. This validates the effectiveness of the CPML absorbing boundary. 

The reflection error of the CPML boundary was quantitatively studied in this paper. Assuming that the excitation source is a plane wave, and the time dependence of the source is

(52) $$g(t)=\exp[\frac{-4\pi {\left(t-t_{0}\right)}^{2}}{t_{1}{}^{2}}]$$

where *t*_0_ and *t*_1_ are constants, and both are equal to 1 × 10^−9^, thus the minimum wavelength of the source is about 0.15 m. The reflection error was computed at an observation point located at cell (30, 30, 30). A reference solution based on an extended lattice was computed in order to isolate the error of the CPML from the grid dispersion error. The relative error was computed as

(53) $$err(\mathrm{dB})=20\log_{10}\frac{\left|E(t)-E^{std}(t)\right|}{\max\left|E^{std}(t)\right|}$$

where *E^std^*(*t*) is the value of the electric field at the observation point computed by the HIE-FDTD method in the extended lattice. *E*(*t*) is the electric field calculated by the HIE-FDTD method truncated by the CPML boundary.

**Figure 2:**
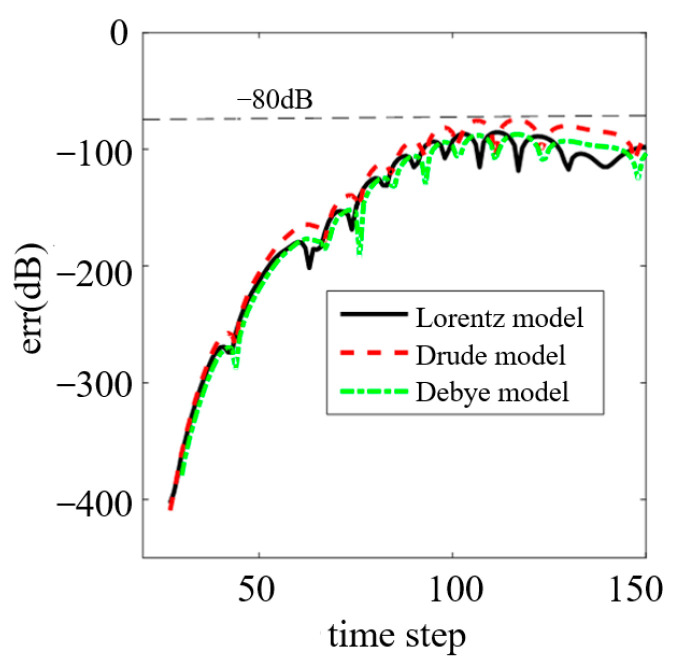
The reflection relative error of CPML in the three dispersion models.

The reflection error with respect to the time step is shown in Figure 2. As can be seen from this figure, the reflection relative error of CPML was less than −80 dB in all three dispersion models. This validates the absorbing performance of the CPML quantitatively.

### 3.2. Accuracy and Efficiency

**Figure 3:**
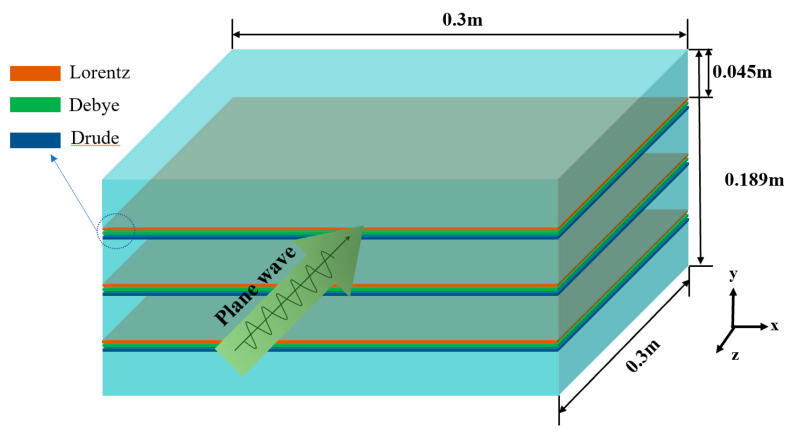
The specific structure of the dispersion plate.

To validate the computational accuracy and efficiency of the dispersion HIE-FDTD method, the scattered field of a multilayer complex dielectric plate was analyzed. The specific structure is shown in Figure 3. A four-layer dielectric plate, whose length, width, and height were 0.3 m, 0.3 mm and 0.045 m, respectively, was separated by three dispersive dielectric layers. The thickness of each dispersive medium layer was 3 mm, and was composed of three kinds of dispersive media: Drude, Debye, and Lorentz, respectively. The parameters of the dispersion media were same as those in Table 1. A plane wave propagating in the -*z* direction was vertically incident on the dielectric plate. The polarization of the electromagnetic wave was along *y* direction, and the time dependence of the wave is shown in Equation (52). Two observation points were located before and behind the plate at 0.03 m and 0.06 m, respectively. A 10-cell thick CPML layer, which was 10 cells far from the plate, was used to terminate all six sides of the lattice. 

**Figure 4:**
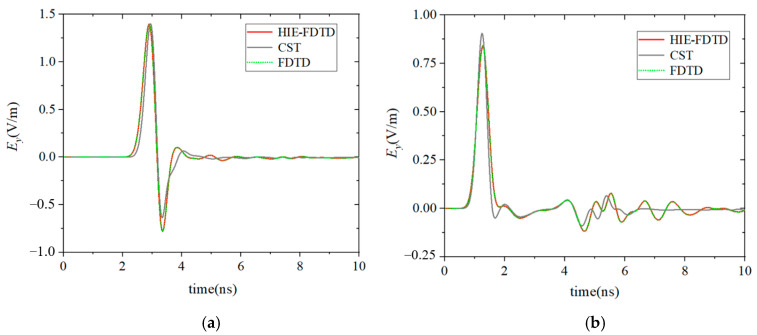
The calculated results of the FDTD method, HIE-FDTD method, and CST software. (**a**) Results of the observation point behind the plate. (**b**) Results of the observation point before the plate.

The electric field *E_y_*(*t*) at the observation points were calculated by using the FDTD method and the HIE-FDTD method. The minimum spatial cell Δ*y*_min_ was 0.0005 m in the dispersion media. In the other computational domain, spatial cells Δ*x* = Δ*y* = Δ*z* = 0.015 m were used. According to the CFL stability condition, the maximum time step size of the FDTD method was 1.66 ps. However, in the HIE-FDTD method, the time step size was not related to the minimum cell size, so its time step size could reach 35.35 ps. The calculated results of these two methods are shown in Figure 4. To validate the accuracy of the methods, the results simulated by using the typical software CST Studio Suite 2018 were also plotted in Figure 4. It can be seen from Figure 4 that the calculated results agreed well with each other.

To complete the simulation above, the time step size of the FDTD method was 1.66 ps, and its total simulation time was 81.7 minutes. In the HIE-FDTD method, the time step size was 35.35 ps, so the total simulation time of the HIE-FDTD method was only 5.4 minutes, which was only 1/15 of that of the FDTD method. This validates that the HIE-FDTD method had a much obvious higher computational efficiency than that of the FDTD method.

**Figure 5:**
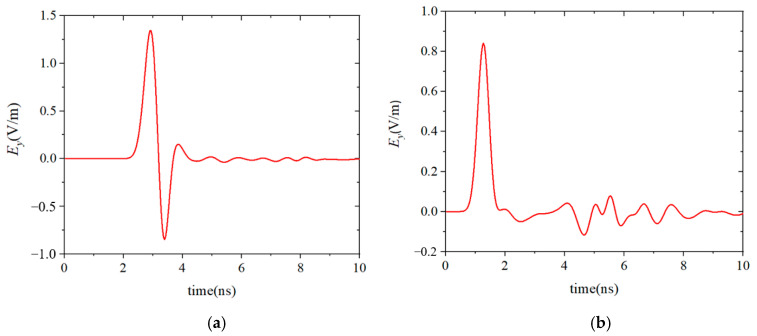
The calculated results of the HIE-FDTD method when the thickness of each dispersive medium layer is 3 nm. (**a**) Results of the observation point behind the plate. (**b**) Results of the observation point before the plate.

For the multilayer complex dielectric plate, as shown in Figure 3, when the thickness of each dispersive medium layer was 3 nm, the electric field *E_y_*(*t*) at the observation points calculated by using the dispersion HIE-FDTD method proposed in this paper is shown in Figure 5.

In this simulation, the minimum spatial cell Δ*y*_min_ was 0.5 nm in the dispersion media. According to the CFL condition, the time step size of the FDTD method was only 1.66 × 10^−6^ ps. It can be deduced that the simulation time of the FDTD method was about 8.17 × 10^7^ minutes, which was about 56736 days. The time step size of the HIE-FDTD method was still 35.35 ps because it was not limited by the grid size in the direction of the media layer thickness, and the simulation time was still 5.4 minutes. It can be seen that the simulation time required by the HIE-FDTD method was much shorter than that of the FDTD method, and the computational efficiency of the HIE-FDTD method was greatly improved.

## 4. Conclusions

In this paper, to solve the dispersive media effectively, the Maxwell equations and three dispersion models were combined, and the hybrid explicit–implicit difference technique was used to obtain the HIE-FDTD method. By introducing the CPML absorption boundary, the method was extended to solve open-domain dispersion problems. Numerical examples were used to verify the absorption performance of the absorption boundary, the calculation accuracy, and calculation efficiency of the algorithm. Since the presented method had a higher computational efficiency than the traditional FDTD method, it could be well applied to solve dispersive media with fine structures such as water, soil, plasma, biological tissue, optical materials, etc.

## Figures and Tables

**Table 1:** Parameters in Drude, Debye, and Lorentz models.

Type	$\varepsilon_{s}$	$\varepsilon_{\infty }$	$\nu_{c}$	$\omega_{0}$	$\omega_{p}$	σ
Drude	/	1	4 × 10^9^	/	2π × 10^9^	0
Debye	2	1	1 × 10^8^	/	/	0
Lorentz	2	1	4 × 10^9^	2π × 10^9^	/	0

## Data Availability

Not applicable.
